# The Platelet Fraction Is a Novel Reservoir to Detect Lyme *Borrelia* in Blood

**DOI:** 10.3390/biology9110366

**Published:** 2020-10-29

**Authors:** Victoria P. Sanderson, Iain L. Mainprize, Lisette Verzijlenberg, Cezar M. Khursigara, Melanie K. B. Wills

**Affiliations:** 1 G. Magnotta Lyme Disease Research Lab, Molecular and Cellular Biology, University of Guelph, Guelph, ON N1G 2W1, Canada; vsande01@uoguelph.ca (V.P.S.); imainpri@uoguelph.ca (I.L.M.); lisette.verzijlenberg@gmail.com (L.V.); 2 Molecular and Cellular Biology, University of Guelph, Guelph, ON N1G 2W1, Canada; ckhursig@uoguelph.ca

**Keywords:** Lyme disease, *Borrelia*, blood processing, EDTA, citrate, platelets, molecular detection, OspA, culture, diagnosis

## Abstract

**Simple Summary:**

To diagnose Lyme disease, a patient’s blood is tested for antibodies that develop as part of the immune response. This can lead to cases being missed or inadequately treated. An ideal test would directly detect the Lyme disease bacteria, *Borrelia*, to provide better clinical guidance. In this study, we aimed to improve the methods currently used to find *Borrelia* in human blood, and identified two opportunities for optimization. We demonstrate that the container most commonly used to collect blood (EDTA) decreases *Borrelia’s* ability to grow, and we identify a superior alternative (citrate). Additionally, using experimentally infected blood, we show that *Borrelia* is highly concentrated in the platelet fraction, making it an ideal candidate for direct detection. These results lay the foundation for diagnostic test development, which could improve patient outcomes in Lyme disease.

**Abstract:**

Serological diagnosis of Lyme disease suffers from considerable limitations. Yet, the technique cannot currently be replaced by direct detection methods, such as bacterial culture or molecular analysis, due to their inadequate sensitivity. The low bacterial burden in vasculature and lack of consensus around blood-based isolation of the causative pathogen, *Borrelia burgdorferi*, are central to this challenge. We therefore addressed methodological optimization of *Borrelia* recovery from blood, first by analyzing existing protocols, and then by using experimentally infected human blood to identify the processing conditions and fractions that increase *Borrelia* yield. In this proof-of-concept study, we now report two opportunities to improve recovery and detection of *Borrelia* from clinical samples. To enhance pathogen viability and cultivability during whole blood collection, citrate anticoagulant is superior to more commonly used EDTA. Despite the widespread reliance on serum and plasma as analytes, we found that the platelet fraction of blood concentrates *Borrelia*, providing an enriched resource for direct pathogen detection by microscopy, laboratory culture, Western blot, and PCR. The potential for platelets to serve as a reservoir for *Borrelia* and its diagnostic targets may transform direct clinical detection of this pathogen.

## 1. Introduction

Lyme disease, a tick-vectored zoonosis caused by a clade of bacteria within the *Borrelia* genus, is a growing public health threat that requires more rapid and sophisticated laboratory diagnostics to mitigate the escalating impact [1,2]. The early stage of the infection can present as non-specific flu-like symptoms as well as an erythema migrans (EM) rash, which was once considered pathognomonic of Lyme [3]. However, the utility of using the EM as a primary diagnostic feature has been challenged by findings of relatively low rash prevalence, variable and inconsistent manifestation [4], difficulty distinguishing the lesion on darker skin tones [5], and the reported appearance of non-borrelial EMs [6,7]. Prompt detection and antibiotic treatment of Lyme in the early stages of disease provides the best prognosis, although symptoms may reoccur and evolve in up to one third of patients [8,9]. Without adequate intervention, *Borrelia burgdorferi* sensu lato (s.l.) (henceforth *B. burgdorferi, Borrelia, or* Bb) can disperse through vasculature and lymphatics to distal locations including the nervous system and heart, giving rise to serious multisystem manifestations that can resemble other diseases [10]. Such mimicry can further delay and complicate diagnosis.

The standard laboratory test for Lyme disease consists of two-tiered serological assessment of IgG and/or IgM response, which suffers both biological and technical limitations [11]. As an indirect test of infection, it relies on the adaptive host immune response, which can be slow to develop, is itself a target of the pathogen, and cannot be used to discern active infection from past exposure [10]. Serological testing is further complicated by microbiological diversity, intrinsic and extrinsic factors that blunt the immune response [12], low-throughput analysis, and inter-lab variability [13].

Alternatively, direct testing techniques such as clinical culture [14], DNA-based methods [15], and antigen detection [16] have long been investigated for their potential to report infection in a sensitive, specific, and timely manner, using accessible biospecimens. To date, however, their application to Lyme disease has been constrained by low and inconsistent spirochetemic burden, *Borrelia* preference for secondary tissue sites, limited understanding of host-adapted *Borrelia* characteristics, and a lack of standardized blood processing methods to recover limited pathogen material in circulation. Depending on the assay, technical challenges can also arise from the dilution effect of host materials, which may obscure the target(s) of interest.

Although blood is an attractive, minimally invasive analyte, estimates of *Borrelia* concentration vary considerably, and appear to depend on the stage of disease, pathogen genotype, pre-analytical methodology, and quantification technique. Vascular burden is anticipated to be highest during the initial dispersal from the tick bite site [17], yet most Lyme *Borrelia* cannot be found using microscopy of peripheral blood [18], and PCR-based DNA detection from serum or plasma during the first month of infection has an average estimated sensitivity range of only 34–62% [19]. This may reflect strain-level differences in propensity for hematogenous dissemination [20], as well as methodological inconsistencies. Few studies have attempted to quantify the number of *Borrelia* per unit volume of blood, but among those that have, the resulting estimates span orders of magnitude. Early work suggested that there were, on average, fewer than 50 *Borrelia* genomes/mL of plasma [21], with a range of less than 20 spirochetes/mL to more than 4000/mL [22]. More recently, a study of early untreated Lyme patients detected a *Borrelia* chromosomal locus by quantitative PCR (qPCR) in 34% of acute patients; within those samples, 4660 copies of the gene were found on average per mL of plasma [23]. Meanwhile, culture-based analyses have estimated 0.1 cultivable organisms/ml of blood in acute disease [24]. In 2016, a new member of the pathogenic Lyme *Borrelia* complex (*B. mayonii*) was discovered that presents with spirochetemia estimated to be 180 times higher than that of conventional *B. burgdorferi* s.l. [18], emphasizing interspecific variability and the clinical relevance of biodiversity. Overall, however, the consensus holds that bacterial load in blood is minor and transient in Lyme disease [2]. Advances in molecular detection technologies suggest that the low concentrations of colonizing *Borrelia* will be surmountable [2,25,26], particularly if the recovery and analysis of the pathogen or its biomarkers can be further optimized.

Here, we used experimentally infected human blood to evaluate the impact of blood collection and processing on *Borrelia* availability and cultivability, and identified that the most common approaches can be detrimental to the organism and the sensitivity of downstream assays. This proof-of-concept work provides an experimental basis for the optimization of blood handling in diagnostic protocols to provide the greatest opportunity for *Borrelia* detection. These methodological improvements can now be applied to the analysis of samples from patients with acute and late disease to determine their impact on assay sensitivity.

## 2. Materials and Methods

### 2.1. Comparison of Clinical Culture Protocols

Studies were compiled by searching PubMed for ((“*Borrelia burgdorferi*”) AND (“culture”)) AND (“blood”) with the requirement that blood-based clinical culture was conducted in the study and both inoculation source and collection tube were clearly indicated. The search was conducted on 15 January 2020, and performed again on 23 June 2020 to capture recent publications. The data collected included the blood components that were used for inoculation of culture, the anticoagulant present in collection tubes for whole-blood and plasma collection, and whether centrifugation steps were implemented for serum and plasma separation from whole blood.

### 2.2. Bacteria

Reference strain *Borrelia burgdorferi* s.s. B31 (ATCC 35210) (referred to as *B. burgdorferi*, *Borrelia*, Bb, and B31 throughout the manuscript) was used to conduct all experiments, except the culture of *Borrelia* from isolated blood fractions where a GFP *Borrelia* strain was used (obtained through Juan Salazar, University of Connecticut). Bacterial stocks were stored at −80 °C in BSK with 20% glycerol. Cultures were propagated at 37 °C, 5% CO_2_ in BSK-H medium with 6% rabbit serum (BB83-500, Dalynn Biologicals, Calgary, AB, Canada; referred to as BSK throughout manuscript). Bacterial culture was always counted using Petroff-Hausser counting chambers under phase-contrast light microscopy.

### 2.3. Vacutainer Anticoaguant Cell Viability Assessments

Ethylenediaminetetraacetic acid (EDTA) exposure was modelled using a 6 mL Becton Dickinson (BD) Biosciences vacutainer K2 EDTA (K2E) 10.8 mg blood collection tube (BD 368661, Becton, Dickinson and Company, Franklin Lakes, NJ, USA). Citrate exposure was modelled using 4.5 mL BD buffered sodium citrate (9NC) 0.105 M = 3.2% blood collection tube (BD 369714, Becton, Dickinson and Company). Serum collection and controls were modelled using 10 mL uncoated BD serum blood collection tube clot activator vacutainer (BD 367820, Becton, Dickinson and Company). In each tube, 5% of the available volume was left unoccupied to provide a microaerophilic environment in accordance with *B. burgdorferi* B31 growth recommendations. The remaining volume was filled with BSK and B31 at a 9:1 ratio. Thus, for EDTA tubes, 0.57 mL B31 culture was added to 5.13 mL BSK. For citrate exposure, 0.428 mL B31 culture was inoculated into 3.85 mL BSK. In uncoated tubes, 0.95 mL B31 was seeded into 8.55 mL BSK or for non-growth controls, and 9.5 mL BSK was added to the uncoated tube. In each biological replicate, a 4–5-day old mid-log phase B31 culture was used to inoculate vacutainers. Vacutainers were loaded using needle and 3-cc syringes to mimic blood draw and maintain vacuum seal. Inoculated vacutainers were then incubated on ice in a Styrofoam container for 48 h to mimic shipping. Following this incubation, vacutainers were opened, and 1 mL was removed and seeded into 6.5 mL of fresh BSK in 8 mL polystyrene round-bottom tubes (Falcon 352027, Corning Life Sciences, Tewksbury, MA, USA). Freshly seeded cultures and vacutainers were then placed at 37 °C, 5% CO_2_ and counted weekly by Petroff-Hausser counting chamber phase-contrast light microscopy (DHC-N01, INCYTO, Republic of Korea). This procedure was conducted in biological triplicate on three separate occasions with a fresh culture each time. Technical replicates were accounted for through Petroff-Hausser counting chamber of five separate squares.

### 2.4. Experimentally Infected Blood Preparation

All experiments involving blood samples were conducted under University of Guelph (UoG) Research Ethics Board (REB) approval number 18-07-007 (amendment approved 1 March 2019 ). After providing informed consent, healthy individuals with ties to the G. Magnotta Lyme Disease Research Lab donated blood for the purpose of protocol optimization and validation. All blood draws were performed by a qualified phlebotomy technician using previously approved venipuncture procedures (UoG SOP014) in the Human Nutraceutical Research Unit clinical trial suite at the University of Guelph. There were no official inclusion criteria for the research study and no fasting requirements. Blood was drawn into sodium citrate vacutainers (BD 369714, Becton, Dickinson and Company) for the purpose of whole-blood collection or uncoated vacutainers (BD 367815, Becton, Dickinson and Company) for serum collection. Blood samples were placed on ice and immediately transported to the lab for inoculation with *B. burgdorferi* B31. To test the impact of centrifugation, B31 was inoculated into whole blood at a 1:1 ratio of culture to whole blood, which resulted in an MOI of 1 Bb:137 RBC. For experiments involving microscopy and molecular analyses of the four blood fractions, 200 μL of whole blood was inoculated with 2 mL BSK alone for uninfected controls or 2 mL of B31 culture in BSK to an MOI of 1 Bb:18.37 RBC. This dilution of blood was intended to improve visualization of blood cell–*Borrelia* interactions. For culture from blood fractions, 1 mL of whole blood was inoculated with 2 mL of *Borrelia* (MOI = 1 Bb:250 RBC). Experimentally infected serum was collected by allowing the spiked blood to clot and subsequently spinning the liquid at 1000× *g*, for 10 min, at 20 °C. Following inoculation of whole blood, experimentally infected and uninfected samples were always incubated for 30 min at 37 °C with a 200 rpm of rotation in a shaking incubator to prevent separation of whole blood before any further processing.

### 2.5. Blood Fractionation by Centrifugation

All centrifugation steps were conducted using a Sorvall Legend XTR centrifuge (Thermo Fisher Scientific, Burlington, ON, Canada) with a Thermo Tx-1000 75,003,017 rotor. Following the incubation step, a portion of infected and uninfected whole blood was set aside, while the remainder was fractionated by standard centrifugation protocols. In the initial experiment testing the direct impacts of centrifugation on stratification of *Borrelia*, one spin was conducted at 400× *g* and 20 °C, for 20 min, to separate cell components (red blood cells and platelets) from liquid plasma. The cell component was diluted 1:1 with 1× PBS (phosphate-buffered saline) for ease of visualization, and this was accounted for in all calculations. Further experiments used two centrifugation steps, allowing the collection of whole blood (pre-centrifugation), a red blood cell-enriched fraction (first pellet), plasma, and a platelet-enriched fraction (second pellet). To separate red blood cells from platelet-rich plasma, whole blood was spun at 120× *g* and 20 °C, for 20 min, from which infected and uninfected red blood cells were collected. Platelet-rich plasma was then separated using a second centrifugation step at 400× *g* and 20 °C, for 20 min, from which infected and uninfected plasma were collected, as well as infected and uninfected platelet pellets (resuspended in 100 μL of PBS) [27]. A portion of each of the collected fractions was stored at −20 °C while the rest was used for additional analyses, as described below.

### 2.6. Immunofluorescent Slide Preparation and Microscopy

Matched infected and uninfected samples for whole blood, red blood cells, plasma and/or platelets were used to prepare slides. Slides were prepared by placing 4 μL of the desired sample onto a microscope slide (16004-382, VWR International, Mississauga, ON, Canada) and smearing with a second slide. Slides were then allowed to air dry for a minimum of 5 min before fixation by dipping in 100% methanol for 30 s. Slides prepared for the initial one-step centrifugation experiment were then mounted with Prolong Gold Antifade with DAPI (Cell Signaling Technology, Inc., Danvers, MA, USA) which contains 4′,6-diamidino-2-phenylindole (DAPI) for DNA detection without any additional staining. These slides were imaged by phase-contrast light microscopy using the Leica DM2000LED microscope (Leica Microsystems, Concord, ON, Canada). Slides prepared for immunofluorescent staining were placed in 1× PBS overnight after methanol fixation and stained the following day. Immunofluorescent staining was conducted using the following steps: slides were blocked in 5% BSA in PBS for one hour, washed twice in 1× PBS, incubated with primary antibody cocktail for 1 h (Platelet IIb/IIIa mouse monoclonal at 1:50 (Santa Cruz Biotechnology, Inc., Dallas, TX, USA) and Acris1002 anti-*Borrelia* rabbit polyclonal at 1:500 in 5% BSA(Origene Technologies, Inc., Rockville, MD, USA) followed by three 1× PBS washes, incubated with secondary antibody cocktail for 1 h (AlexaFluor 555 goat anti-mouse 1:500, AlexaFluor 488 donkey anti-rabbit 1:200 in 5% BSA (A-21424 and A-11029, ThermoFisher Scientific, Burlington, ON, Canada)) and mounted with Prolong Gold Antifade with DAPI. Slides were imaged by phase-contrast light microscopy and fluorescence on Leica DM550B. Fluorescent and phase-contrast images were combined into composites using ImageJ. Three smears were conducted for each of the three biological replicates and three images were captured for each slide. Counting of total cells and interactions between cells was conducted using the ImageJ CellCounter plugin (https://imagej.nih.gov/ij/plugins/cell-counter.html) to produce average technical and biological replicates.

### 2.7. Culture from Blood Fractions

Experimentally infected platelet and serum fractions were collected as described above, and then 1 mL of the sample was inoculated into 6.5 mL BSK in 8 mL polystyrene round-bottom tubes (Falcon 352027). Cultures were incubated at 37 °C, 5% CO_2_, and counted weekly. Tubes were always inverted prior to counting.

### 2.8. Western Blotting

Experimentally infected and uninfected samples of each fraction were prepared by adding 16 μL of the sample to 160 mL of lysis buffer (10% glycerol, 50 mM Hepes pH 7.5, 150 mM NaCl, 1.5 mM MgCl_2_, 1 mM EGTA, 10 mM NaPPi, 100 mM NaF, 1% Triton X-100 plus ProteaseArrest (786–108, G-BioSciences, St. Louis, MO, USA) and PhosphataseArrest (786–450, G-BioSciences) and then incubating at room temperature for 20 min. Following lysis, samples were centrifuged at 12,000× *g* and 4 °C, for 5 min, and the supernatant was frozen at −20 °C, subsequently referred to as the fraction lysate. After, 5 × SDS loading buffer (250 mM Tris-HCl, pH 6.8, 10% SDS, 30% glycerol, 0.1% Bromophenol Blue, 0.5% beta-mercaptoethanol) was added to the fraction lysate at a 1:4 ratio. A volume of 20 μL of the sample was then loaded into the wells of a 12.5% SDS-PAGE gel. Electrophoresis was conducted using a BioRad Mini Protean Electrophoresis System Bio-Rad Laboratories (Canada) Ltd. (Mississauga, ON, Canada) with the following parameters: 50 V, 20 min then 150 V, 50 min. The gel was then placed in transfer buffer (3 g/L Tris base, 14.4 g/L glycine, dH_2_O, 25% methanol) for 15 min. A PVDF membrane (TM300-0.45PVDF, FroggaBio, Concord, ON, Canada) was activated in methanol and placed in water. Transfer was conducted on the Pierce^TM^ Power Blotter (Thermo Fisher Scientific, Burlington, ON, Canada) with the following parameters: 0.8 A, 25 V, 7 min. Following protein transfer, the membrane was blocked with 5% skim milk in TBS (Tris-buffered saline) overnight. The membrane was then washed briefly in TBS-T (TBS + 0.1% Tween-20) then incubated with the primary antibody for 3 h (SC58093 OspA mouse monoclonal, Santa Cruz Biotechnology, Inc., Dallas, TX, USA). The membrane was washed 3 times in TBS-T for 10 min each. A volume of 2 μL ECL anti-mouse IgF, HRP-linked secondary antibody (Cytiva, Global Life Sciences Solutions Canada ULC, Mississauga, ON, Canada) was added to 20 mL of 5% skim milk. Secondary antibody incubation was conducted for 1 h. The membrane was washed in TBS-T for 10 min each. All original western blots can be found in Figure S1.

### 2.9. Polymerase Chain Reaction (PCR)

Each PCR reaction was prepared in 0.2 mL single PCR tubes with 16.4 μL PCR water, 2.5 μL 10× reaction buffer, 2 μL 25 mM MgCl_2_, 0.5 μL dNTPs, 0.1 μL Taq DNA polymerase (ThermoFisher-EP0402 recombinant 5 U/μL), 1.25 μL 10 μM forward primer, 1.25 μL 10 μM reverse primer, and 1 μL template. Primers amplified the variable region of the 16S rRNA gene. Primer sequences were as follows: Primer 1 ATGCACACTTGGTGTTAACTA and Primer 2 GACTTATCACCGGCAGTCTTA [28]. Template DNA was unprocessed (no DNA isolation) and directly added as the experimentally infected blood fraction and the PCR reaction was run with recommended conditions. Each amplified sample was then separated on a 1% ultra-pure agarose gel with loading buffer consisting of 6× DNA loading buffer (30% glycerol, 0.25% orange G dye). The gel was stained with 3× RedSafe for 30 min, and imaged by UV illumination.

### 2.10. Statistical Analyses

All statistical analyses were conducted using Prism8 (GraphPad Software, San Diego, CA, USA).

## 3. Results

### 3.1. Appraisal of Existing Clinical Culture Practices

**Figure 1:**
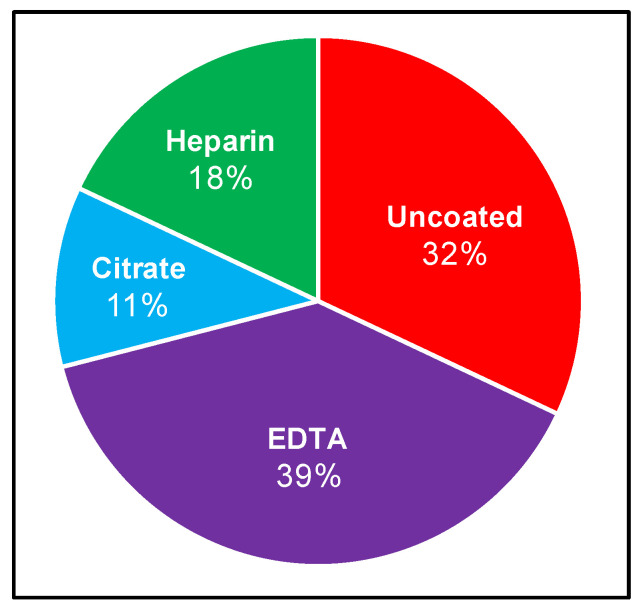
Reported use of anticoagulants to recover *Borrelia* from human blood. Relevant literature was curated (as described in Materials and Methods) to compare which vacutainers are commonly used when conducting *Borrelia* clinical culture from patient samples. Percentage calculated as number of times the vacutainer was used relative to the total experiments conducted using vacutainers in a set of applicable studies.

Although the recovery of *Borrelia* from patient samples has routinely been attempted for several decades, no standardized protocol exists. We therefore set out to curate the LD direct detection literature to evaluate blood collection and processing methods. Specifically, we sought to identify the range of approaches used and the strength of evidence upon which these methodological choices are based, and to determine whether the field is converging on a consensus strategy. The stepwise process of drawing blood for direct testing begins with venipuncture and blood collection into a vacutainer, which is either uncoated (yielding serum), or treated with one of EDTA, sodium citrate, or heparin anticoagulants, from which plasma and various cell fractions can be obtained. A systematic review of NCBI-indexed literature relating to *B. burgdorferi* blood culture allowed for compilation of inoculum sources, anticoagulants, and centrifugation steps for subsequent analysis. These results are summarized in Figure 1 and Table A1, and reveal that *Borrelia* clinical culture protocols have varied widely [9,14,22,29,30,31,32,33,34,35,36,37,38,39,40,41,42,43,44,45,46]. Blood or skin biopsy from EM rash are the most common inoculum sources, and although the latter is informative in a subset of early infection cases, it is largely not applicable to ongoing disease. Within the cohort of studies that inoculate from blood, there is also a range of blood components used, with serum (9/28 relevant experiments) and plasma (13/28 relevant experiments) being the most common. These 28 experiments correspond to the 21 independent studies presented in Table A1, as several studies use multiple parallel inoculum sources. Culture from plasma reportedly leads to a greater chance of recovery compared to serum [29,34,42,47]. A small number of studies have incorporated cellular components or whole blood [9,22,42]. The most common anticoagulant vacutainer used for whole-blood and plasma collection is EDTA (11/28 relevant experiments) followed by citrate (3/28 relevant experiments), and although several studies used heparin in the 1990s (5/28 relevant experiments), this trend has not continued. Although one relatively recent investigation, which scored *Borrelia* recovery from a mixture of blood from spirochetemic SCID mice and healthy humans, suggested that EDTA was not ideal for this purpose [48], the anticoagulant remains in use. Once blood has been collected, some studies allow for natural clotting (serum) or separation (plasma), while others centrifuge blood to obtain the desired component [29,34]. At least 60% of studies implemented centrifugation with speeds ranging from 100× *g* to 1100× *g* for the purpose of either serum or plasma separation. Following inoculation, cultures are typically monitored at a particular interval using dark-field microscopy and then confirmed using some combination of molecular techniques such as PCR (with or without sequencing), immunostaining, and high-resolution microscopy. The time frame within which cultures are monitored varies significantly, ranging from 4 to 16 weeks, with or without subcultivation.

Overall, there is no consensus approach to *Borrelia* recovery, and the procedural differences have made it challenging to compare studies, draw conclusions about optimal techniques for clinical detection, or reconcile incongruent results in Lyme disease patient samples [2]. The most common approaches include the use of plasma, collected using EDTA, and processed with centrifugation, although robust evidence supporting these choices is lacking. It was therefore of interest to evaluate the effectiveness of common approaches, and provide an experimental basis for the standardization and optimization of blood handling for *Borrelia* detection.

### 3.2. Effect of Anticoagulants on Borrelia Viability

Of the three anticoagulants that have been used for *Borrelia* recovery, heparin was eliminated from our experimental design at the outset due to previously documented contraindications. It is a known inhibitor of molecular analyses, particularly PCR, and is also a well-characterized ligand of *Borrelia* [49,50], which could further confound observations. Perhaps as a consequence, heparin has fallen out of use in Lyme disease investigations since the late 1990s (see Table A1). There are precedents in the literature to suggest that all anticoagulants used for recovery of whole blood may impact growth to a certain degree, but the relative inhibition of growth at the microbiological level has not yet been assessed [48]. In this experiment, *B. burgdorferi* strain B31 cultured in liquid BSK medium was seeded directly in the EDTA, citrate, and uncoated vacutainers, and either grown in that environment or subcultured in fresh broth and counted weekly using a Petroff-Hausser chamber.

**Figure 2:**
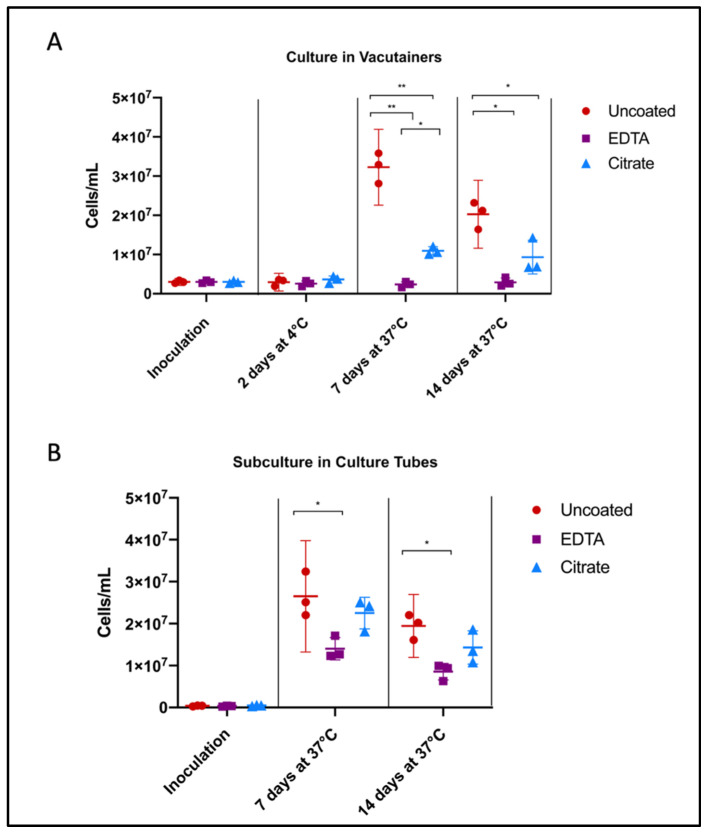
Influence of anticoagulants on *Borrelia* cultivability. To consider anticoagulant impact on *Borrelia* culture growth, we performed cell counts following inoculation of *Borrelia* into BSK broth and incubation in uncoated, EDTA, and citrate vacutainers, monitoring (**A**) the direct effects of anticoagulants on pathogen growth, and (**B**) consequences upon subcultivation. Culture was aliquoted into fresh BSK in an uncoated culture tube after 48 h in vacutainers (from A), to mimic standard shipping and laboratory culture procedure. Horizontal lines represent mean of three biological replicates and whiskers display the standard deviation. Asterisks indicate a significant difference (* = *p* < 0.05, ** = *p* < 0.0001) by one-way ANOVA and Tukey’s multiple comparisons post-hoc test (*n* = 3).

Uncoated/unaltered culture reached peak growth by 7 days, followed by the stationary/death stage. Citrate- and EDTA-exposed cultures followed similar growth trends, but were unable to reach the same maximum concentration of cells. After 7 days of growth directly in the vacutainer environment, *Borrelia* cultures in citrate tubes achieved a higher cell concentration than those in EDTA (*p* = 0.0103) (Figure 2A). Similarly, after subculture, *Borrelia* inoculated from citrate vacutainers recovered to within range of the unaltered cultures, reaching 85% of the positive control growth (*p* = 0.4934) while *Borrelia* exposed to EDTA only reached 53% of the uncoated positive control (*p* = 0.0221) (Figure 2B).

### 3.3. Fraction Enrichment of Borrelia during Blood Processing

Serum and plasma are most commonly used as the inoculation source for clinical culture, yet few studies have ventured into testing whole blood by molecular techniques. Our goal was therefore to evaluate whether the liquid component of blood is truly the best source of *Borrelia*, in order to identify the optimal blood fraction for direct detection purposes.

**Figure 3:**
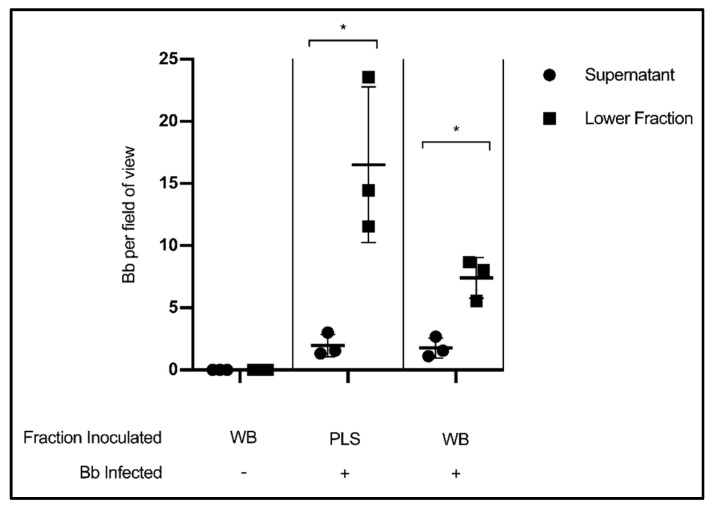
Stratification of *Borrelia* in blood following separation of cells and plasma. Quantification of *Borrelia* per field of view in the supernatant/liquid fraction and cellular/lower fraction following centrifugation at 400× *g*, 20 min. Data presented as the means with standard deviation. Asterisks (* = *p* < 0.05) indicate significance difference using one-tailed paired t-tests (*n* = 3).

In this experiment, citrated whole blood (WB) collected from healthy volunteers was experimentally infected with *Borrelia*, incubated for thirty minutes, then centrifuged according to standard blood processing procedures to separate plasma from a cellular fraction consisting of erythrocytes, leukocytes and platelets. The results show that the liquid component of blood had far fewer *Borrelia* per field of view than the lower, cellular fraction (*p* = 0.0286) (Figure 3). This finding indicates that valuable spirochetes are being separated into the host cell pellet rather than the plasma so often used for testing. Uninfected whole blood was included as a microscopy control. As an additional control, cleared plasma (PLS) was experimentally infected with *Borrelia*, and also showed more spirochetes per field of view in the lower fraction than the supernatant (*p* = 0.0318).

**Figure 4:**
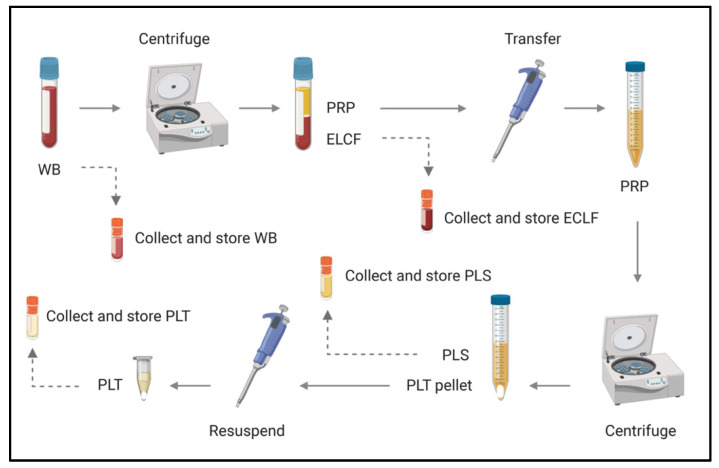
Schematic of blood fractionation protocol. Multiple steps were implemented to obtain a panel of blood components for comparison by microscopy, culture and molecular analyses. The initial centrifugation step pellets red and white blood cells at 120× *g* for 20 min, leaving a platelet-rich plasma supernatant which is subsequently spun at 400× *g* for 20 min to obtain platelets (pellet) and acellular plasma (supernatant). WB = whole blood, ELCF = erythrocyte, leukocyte cellular fraction, PRP = platelet-rich plasma, PLS = plasma (platelet poor), and PLT = platelet fraction.

With the understanding that *Borrelia* partitions with host cells, it was of interest to pursue subsequent investigations of different blood fractions to identify where the greatest enrichment occurs. Whole blood was collected as above, and inoculated with *B. Burgdorferi* (Bb) at a relatively conservative multiplicity of infection (MOI) of 1 Bb:18.37 erythrocytes, in recognition of the low spirochetemic burden observed in disease. The blood was then separated into erythrocyte-leukocyte cellular fraction (ELCF), platelet-rich plasma (PRP), plasma (PLS) and platelet (PLT) fractions using the protocol depicted in Figure 4. Microscopy, culture, and molecular analyses were used to assess the fractions for *Borrelia* enrichment.

**Figure 5:**
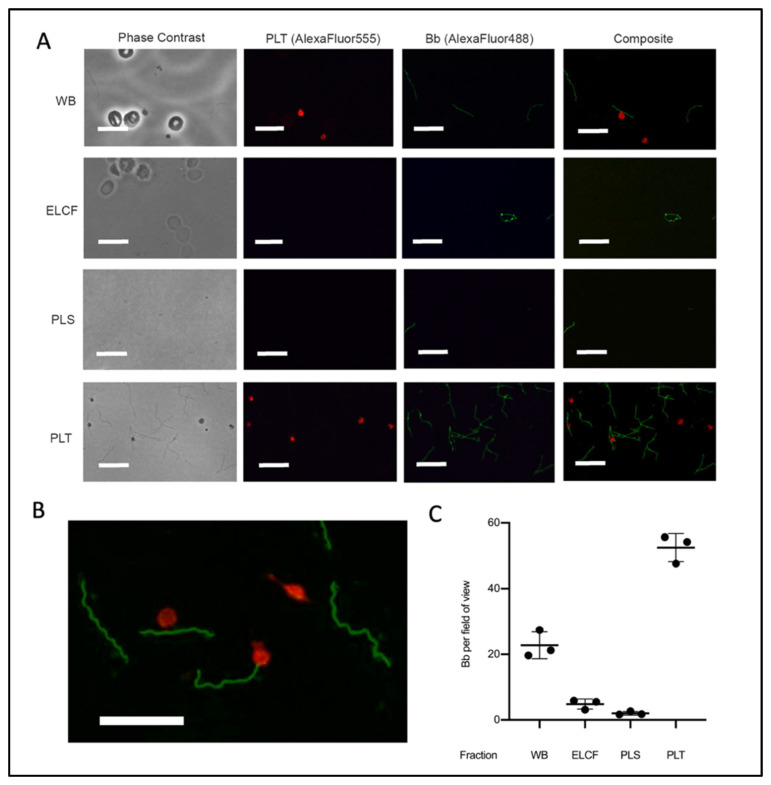
Microscopy of experimentally infected blood before and after fractionation. (**A**) Representative immunofluorescent micrographs of *Borrelia* (green) in fractions collected from experimentally infected whole blood (platelets labelled red). Scale bar = 13 μm. (**B**) Magnification of PLT fraction composite demonstrating co-localization. Scale bar = 13 μm. (**C**) Corresponding quantification of *Borrelia* per field of view from A; *n* = 3. All comparisons by ANOVA and Tukey’s multiple comparisons post-hoc test were significant with *p* < 0.005 except red blood cells and plasma. Data represented as the means with standard deviation. WB = whole blood, ELCF = erythrocyte, leukocyte cellular fraction, PRP = platelet-rich plasma, PLS = plasma (platelet poor), and PLT = platelet fraction.

Immunofluorescent microscopy allowed for specific labelling of *Borrelia* (green) and platelets (red) (Figure 5A,B). The results of this investigation suggested that the platelet fraction consistently and drastically had the most *Borrelia* per field of view (Figure 5C). Co-localization between platelets and *Borrelia,* which we defined as direct juxtaposition of the two cell types, was observed in micrographs from both whole blood and platelet fractions. Enumeration of these co-localization events showed that 20.3% and 25.9% (*p* = 0.338, two-tailed *t*-test) of platelets have *Borrelia* co-localized in whole blood and the platelet-enriched fraction, respectively (Table 1). Additionally, the spirochetes were noted to co-localize with erythrocytes in whole blood [51].

**Figure 6:**
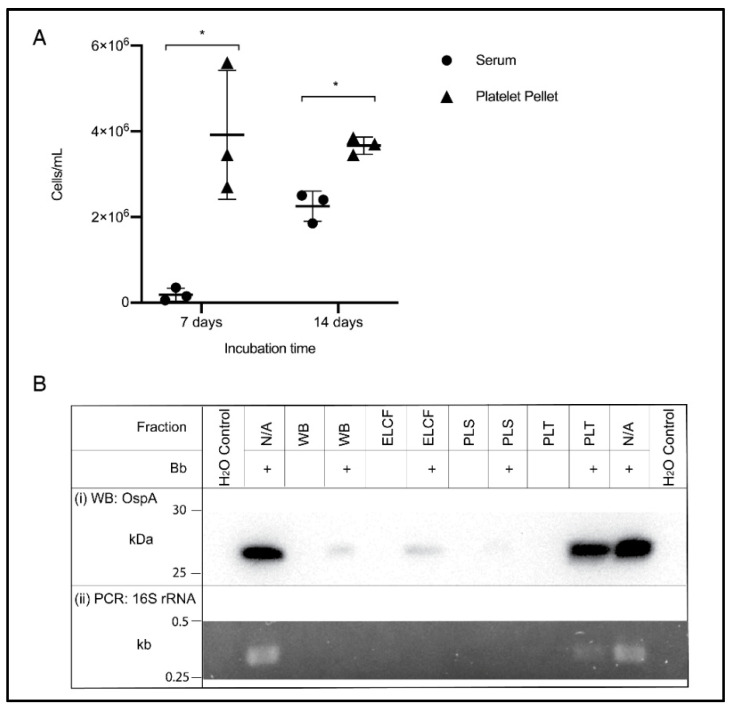
Culture and molecular detection of *Borrelia* in blood fractions. (**A**) Broth culture of serum and platelet fractions collected from experimentally infected blood samples, monitored weekly for growth. Data represented as the means with standard deviation. Asterisks indicate significant difference by one-tailed paired *t*-test (* = *p* < 0.05) (*n* = 3). (**B**) Detection of OspA protein by Western blot (i) and the 16S rRNA gene by PCR (ii) in experimentally infected and matched uninfected blood, and controls. WB = whole blood, ELCF = erythrocyte, leukocyte cellular fraction, PLS = plasma, and PLT = platelet fraction.

The ability to culture from blood is an example of a relevant downstream application of optimized blood processing methods, and the accepted gold standard for detection of *Borrelia* infection. Based on the results of the previous experiment, the culture of *Borrelia* from the platelet fraction was compared to serum, a literature standard. Cultures were also inoculated using whole blood and ELCF; however, after one week, no growth could be visualized due to the highly heterogenous nature of the blood sample [51]. Additionally, PLS and PRP inoculated cultures showed no growth after 7 days and thus only serum was retained as the comparison to the prolific platelet pellet. As shown in Figure 6A, the platelet fraction displayed higher concentrations of spirochetes at both 7 (*p* = 0.0279) and 14 days (*p* = 0.0034) compared to the commonly used serum inoculum. Also of note, at 7 days, *Borrelia* were highly motile in the platelet culture, while the few cells visible in the serum culture were not, but by 14 days, there were motile spirochetes in both fractions.

An additional downstream application and method for comparing these blood fractions is through molecular analyses. Western blot analysis of protein content in each fraction using an OspA monoclonal antibody shows a strong band in the experimentally infected platelet fraction, comparable to the positive control of cultured *Borrelia* lysate (Figure 6(Bi)). Faint bands can be seen in the experimentally infected WB and ELCF and a very faint, barely visible band, can be seen in the infected plasma fraction. Diagnostic PCR specific for the 16S rRNA gene of *B. burgdorferi* was attempted for each fraction without any DNA isolation, and the platelet fraction was the only sample, aside from the positive control lysate, to generate a visible band by agarose gel electrophoresis (Figure 6(Bii)).

## 4. Discussion

The experimentally infected human blood system evaluated in this study demonstrates that blood processing substantially influences the ability to recover and detect *Borrelia*. Methodological choices in the literature are often unfounded, and there is a lack of consensus between studies. Our findings establish that the frequently used anticoagulant (EDTA), and routine inoculum sources (plasma and serum), are not ideal for applications that directly detect *Borrelia*. As previously suggested, sodium citrate anticoagulant is superior to EDTA for the recovery of *Borrelia*, leading to a greater peak culture density. Routine fractionation of blood into plasma and a cell pellet segregates *Borrelia* into the cellular fraction, leaving the plasma with few spirochetes. A deeper analysis of multiple blood fractions using microscopy, culture, and molecular techniques identified that the platelet fraction obtained through centrifugation is highly concentrated with *Borrelia*, providing a novel reservoir of detectable *Borrelia* targets in the blood. Together, these findings identify opportunities for protocol optimization and provide refined guidelines for direct detection.

The risk of losing or altering *Borrelia* begins the moment blood is drawn into a vacutainer. Previous studies suggested that EDTA is inferior to citrate, potentially due to its stronger calcium and magnesium chelation properties [48]. One study of *Borrelia miyamotoi*, a species related to tick-borne relapsing fever pathogens, found the minimum inhibitory concentration of EDTA to be 0.25 mg/mL, while the nominal concentration in an EDTA vacutainer is 1.8 mg/mL [52]. Our results provide additional evidence that EDTA inhibits *Borrelia* growth, while demonstrating the extent to which a clinically relevant workflow impacts cultivability. In clinical samples that already have a low concentration of spirochetes, reducing growth capacity by roughly half through EDTA exposure would severely decrease the ability to obtain positive culture results. Sodium citrate reached 85% of normal growth in our assessment, and therefore maximizes recovery from whole-blood components. These results are also relevant for molecular analyses of viable, minimally processed spirochetes, as appropriate handling ideally limits antigenic variation and metabolic shifts [53]. Ultimately, reducing procedural artifacts and preserving the integrity of the pathogen provides the greatest opportunity to obtain accurate direct test results.

Subsequent steps of direct testing from blood often involve separation of whole blood to obtain the desired blood component, typically plasma or serum. Our analysis of serum as an inoculum did not show appreciable growth until 14 days, indicating the starting concentration was either very low, or spirochetes were affected by the clotting process. It has been hypothesized that the coagulation process used to generate serum may trap spirochetes within the clot, *Borrelia* may bind to activated platelets, or substances could be released that adversely impact bacteria [34]. Inoculating with plasma avoids the negative impacts of clot formation, and reportedly yields more positive culture results compared to matched serum [34]. However, our results show that the vast majority of spirochetes are concentrated in the cellular component after processing experimentally infected whole blood to recover plasma, suggesting that plasma is also not an ideal inoculum source. It should be noted, however, that reports of plasma-based analysis often fail to distinguish between platelet-rich and cleared plasma. Based on our findings, we anticipate that this distinction could impact recovery considerably. Nevertheless, cellular fractions have rarely been investigated in the literature as *Borrelia* reservoirs. Our observations demonstrate that centrifugation as low as 400× *g* contributes to the concentration of spirochetes, which was a surprising result as 1000–8000× *g* forces are typically used to pellet *Borrelia* from *in vitro* culture [54,55,56]. Gravitational separation improved the yield of *Borrelia* DNA in one study [28], and may represent an alternative to centrifugation to limit *Borrelia* loss during liquid recovery. However, co-localization was observed between platelets and *Borrelia* (Table 1), as well as erythrocytes and *Borrelia* [51]. As a result of these interactions, a proportion of spirochetes may associate with platelets, erythrocytes, and even lymphocytes, separating into the cellular fraction regardless of centrifugation [57]. Yet, examination of individual fractions revealed that putative red blood cell interactions did not result in a robust pathogen yield according to cell counts (Figure 5) or molecular target detection (Figure 6B) in the erythrocyte and leukocyte cell pellet, which contains approximately 600 red blood cells for every 1 white blood cell in the average human. It was likewise not possible to obtain a quantifiable *Borrelia* culture from whole blood or the ELCF due to the abundance of host material. Therefore, any marginal improvement in *Borrelia* recovery achieved by generating a single heterogeneous cell pellet (as in Figure 3) is offset by the considerable dilution effects from the host cells. Meanwhile, the consistent platelet–*Borrelia* co-localization dynamics observed in whole blood and the separated platelet fraction support the hypothesis that *Borrelia* enrichment observed in the platelet fraction could be partly mediated by host cell associations. A few studies have previously considered *Borrelia*–platelet interactions, and concluded that *Borrelia burgdorferi* (sensu stricto), *Borrelia garinii,* and *Borrelia afzellii* as well as relapsing fever (RF)-causing *Borrelia hermsii* are capable of associating with activated platelets [17,58,59]. Host–pathogen interactions with various blood cells may help to identify where *Borrelia* are found within the blood.

Indeed, multiple modes of analysis, including immunofluorescent microscopy, culture and molecular detection, corroborate that the platelet fraction is enriched with *Borrelia* in our experimental infection model. This observation identifies for the first time that the platelets isolated by two-step centrifugation could provide a reservoir of detectable targets with immense opportunity for further exploration. Early studies in rats and humans briefly alluded to the use of platelet-rich plasma as a superior source of host-derived *Borrelia* [17,22], although this observation was never investigated in more detail. The distribution of spirochetes between blood fractions has also been considered in the context of RF *Borrelia* and blood transfusions [60]. In this study, Thorp and Tonnetti found the majority of RF spirochetes within the red blood cell fraction; however, they diluted the platelet fraction substantially. Their findings nevertheless support the conclusion that the liquid fraction of blood is not an ideal source for direct testing.

The platelet fraction similarly displays promise as a culture inoculum, presenting with a highly motile culture at higher peak concentration than serum in a much shorter time span of only 7 days. For culture it is particularly important to obtain high concentrations of spirochetes as 5 × 10^4^ cells/mL is the lower limit for enumeration via Petroff-Hausser counting chamber. Despite these potential advances in growth rate, culture will likely still be too slow and potentially too insensitive for clinical utility but remains a valuable research tool. Comparatively, PCR can be a much more sensitive technique that can reportedly detect as low as 10–100 copy numbers and has been used in Lyme disease research for *Borrelia* detection in blood [61]. The majority of PCR-based studies test serum or plasma, but as the results of this study show, the enriched platelet fraction could further improve PCR detection. Of the available blood cell fractions, enrichment of *Borrelia* with platelets is particularly valuable in the diagnostic context because they have limited quantities of eukaryotic DNA, RNA [62], and inhibitory substances, which can dilute molecular pathogen targets and/or interfere with PCR amplification in the leukocyte and erythrocyte fraction [63]. Research on *Borrelia* protein content in blood is limited, but our analysis demonstrates that outer surface protein detection by Western blot is possible with the concentrated platelet fraction. Regardless of the target, the challenges facing direct detection may be ameliorated, at least in part, by probing the enriched fraction using various tools that have come to fruition across the Lyme disease literature.

Although the outcomes of our studies are clear under the conditions tested, *Borrelia* are diverse and complex bacteria, and their associations with the host are incompletely characterized. Parameters that could be varied to further contextualize these findings include the origin and derivation of *Borrelia* strains used (lab-adapted vs. clinically isolated), centrifugation speeds, infection time, platelet count, and bacterial MOI in blood. The highest MOI used in this study was 1 Bb:20 erythrocytes for experimental infection of whole blood, which is significantly lower than other host–cell interaction studies. However, this inoculation strategy resulted in a ratio of 2.3 Bb:1 Plt in whole blood and in the platelet fraction after centrifugation. Studies investigating host-pathogen associations typically have more *Borrelia* present than host cells, spanning MOIs of 5 spirochetes:1 host cell on the low end of the spectrum, up to 5000 *Borrelia*/host cell [64]. Increasing the pathogen burden and/or incubation length in the infection model could result in a number of physical and physiological changes that alter how *Borrelia* partition in the blood, potentially enhancing the phenomenon we describe here. Ultimately, evaluating the *Borrelia* content of platelets obtained from naturally infected LD patients would provide the most relevant insight into the clinical applicability of this blood fraction.

## 5. Conclusions

This research provides foundational work in the field of blood collection and processing for direct detection of *Borrelia* in blood. Questioning the seemingly benign methodological choices led to the discovery that the platelet fraction collected from citrated whole blood is a *Borrelia*-enriched reservoir. These findings should be used both to re-interpret past studies with suboptimal methodology, and to guide forthcoming research. Future studies should consider the implications of each methodological choice involved in blood collection, processing, and testing. The findings presented here advance our understanding of direct pathogen detection, and may contribute to faster, more reliable, and clinically useful diagnostic tools for Lyme disease.

## Figures and Tables

**Table 1:** Co-localization of *Borrelia* and platelets in experimentally infected whole blood and the platelet-enriched fraction obtained through centrifugation. Data collected as average counts per field of view with standard deviation from three biological replicates. Bb: *B. burgdorferi*; Plt = platelet.

Component	Avg Bb	Avg Plt	Bb-Plt/Total Bb	Plt-Bb/Total Plt
Whole Blood	22.8 ± 4.1	9.9 ± 2.7	8.9 ± 2.0%	20.3 ± 5.4%
PLT Fraction	53.9 ± 4.7	22.6 ± 14.8	11.3 ± 5.6%	25.9 ± 7.1%

## Supplementary Materials

The following are available online at https://www.mdpi.com/2079-7737/9/11/366/s1, Figure S1: original western blot figures.

## Appendix A

**Table A1:** Summary of methods used in Lyme disease clinical culture studies. Consideration was given to which blood components were used for inoculation of culture, what anticoagulant was present in collection tubes for whole blood and plasma collection and whether centrifugation steps were implemented for serum and plasma separation from whole blood. Data compiled by searching ((“*Borrelia burgdorferi*”) AND (“culture)) AND (“blood”) with the requirement that blood-based clinical culture was conducted in the study and both inoculation source and collection tube were clearly indicated. Colour coding represents collection tube (red: uncoated, purple: EDTA, blue: citrate, and green: heparin) and studies were ordered chronologically to present vacutainer usage over time. Literature search was conducted on 15 January 2020, and updated on 23 June 2020 using PubMed.

Inoculation Fraction	Collection Tube	Centrifugation	Citation
Plasma	EDTA	Unspecified	Horn et al. 2020 [40]
Serum (+ a few blood cells)	Uncoated	Yes (low speeds)	Middelveen et al. 2018 [9]
Serum (+ a few blood cells)	Uncoated	Yes (low speeds)	Middelveen et al. 2015 [41]
Serum and whole blood	Uncoated, EDTA and direct into BSK	No	Sapi et al. 2013 [42]
Plasma	EDTA	Yes (260× *g*, 15 min)	Liveris et al. 2012 [43]
Plasma	EDTA	Yes (260× *g*, 15 min)	Liveris et al. 2011 [44]
Plasma	Citrate	Yes (800 rpm, 10 min)	Maraspin et al. 2011 [45]
Plasma	Citrate	Yes (100× *g*, 5 min)	Cerar et al., 2008 [46]
Plasma	EDTA	Yes (350× *g*, 15 min)	Coulter et al. 2005 [29]
Plasma	EDTA	Unspecified	Wormser et al. 2005 [30]
Plasma	EDTA	Yes (260× *g*, 15 min)	Wormser et al. 2001 [31]
Plasma	Citrate	Yes (800 rpm, 5 min)	Arnez et al. 2001 [32]
Plasma	EDTA	Unspecified	Marques et al. 2000 [33]
Serum and plasma	Uncoated and EDTA	Yes (260× *g*, 15 min)	Wormser et al. 2000 [34]
Serum, plasma and whole blood	Uncoated and heparin	Unspecified	Liveris et al. 1999 [35]
Whole blood	EDTA	No	Phillips et al. 1998 [36]
Serum and whole blood	Heparin	Yes (serum—1100× *g*, 10 min)	Wormser et al. 1998 [37]
Serum, whole blood, plasma, buffy coat and erythrocytes	Uncoated and EDTA	Yes (260× *g*, 15 min)	Goodman et al. 1995 [22]
Whole blood	Uncoated (directly into BSK)	No	Berger et al. 1994 [38]
Serum and whole blood	Uncoated and heparin	No	Wallach et al. 1993 [39]
Serum and whole blood	Uncoated and heparin	Yes (serum—500× *g*, 10 min)	Nadelman 1990 [14]
