# Supplementary material for: The Platelet Fraction Is a Novel Reservoir to Detect Lyme Borrelia in Blood
Source: Biology (Basel). 2020 Oct 29;9(11):366. doi: 10.3390/biology9110366 (PMC7694117; doi:10.3390/biology9110366)
Supplement: Supplementary file 1 [file biology-09-00366-s001.pdf]

The Platelet Fraction is a Novel Reservoir to Detect Lyme Borrelia in Blood

Sanderson et al.

Figure 3B Full Unedited Western Blot and Gel

3B i) WB; Anti-OspA (SC58093 Mouse Monoclonal)

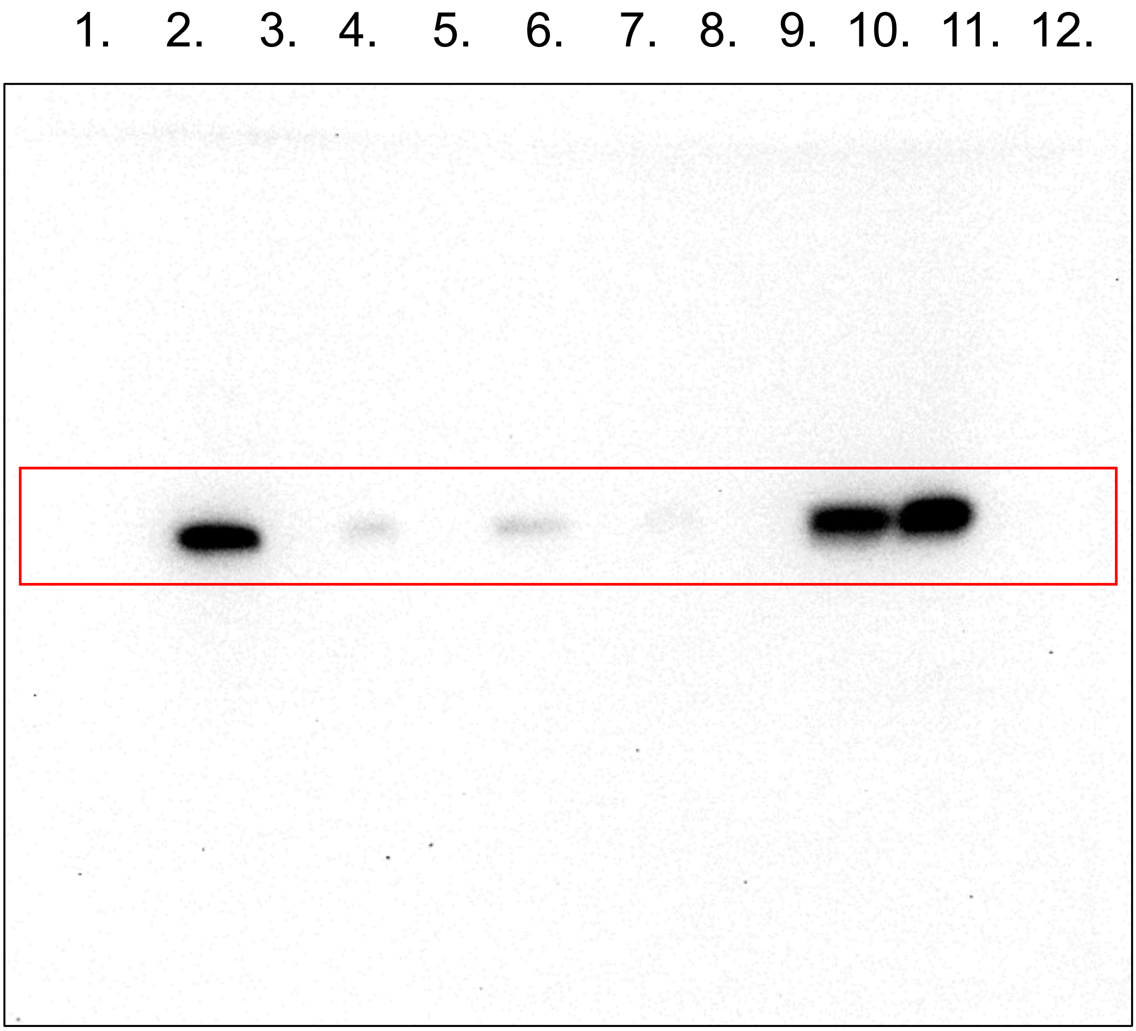

Original perimeter – Black

Cropped region - Red

3B ii) PCR; 16S rRNA gene (primers provided in methods)

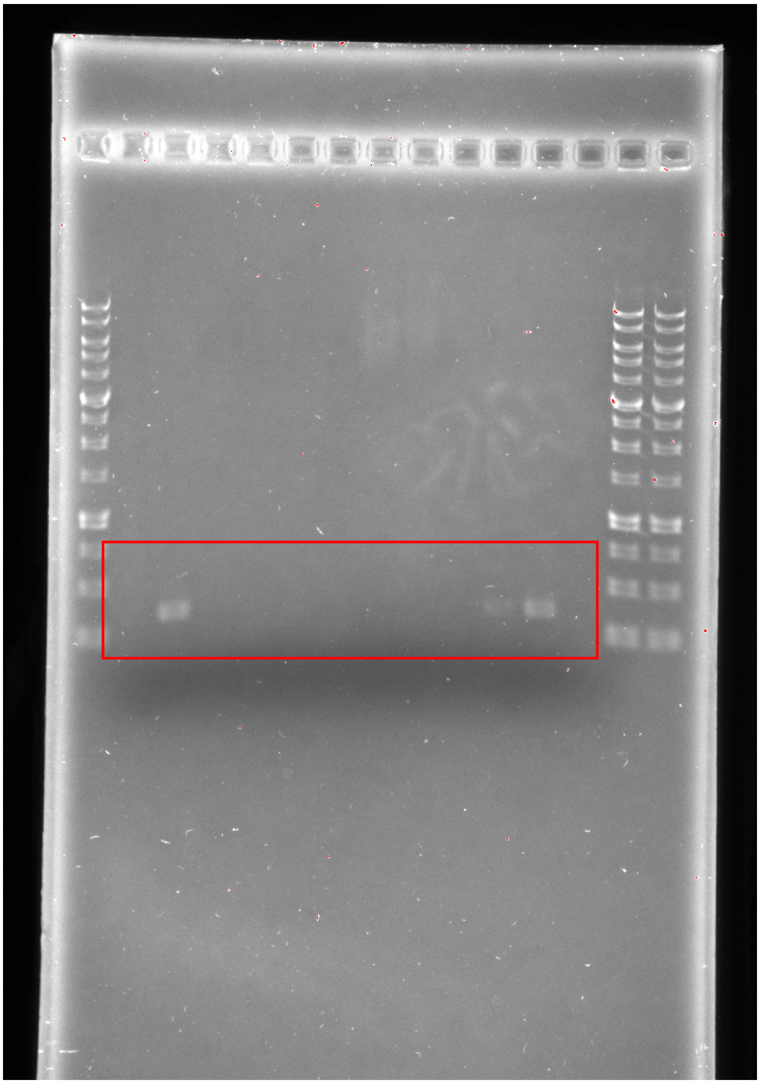

Cropped region - Red
